# Spatial and Economic Proximity of Cigarette Sales to School Children in Mongolia

**DOI:** 10.21203/rs.3.rs-4088408/v1

**Published:** 2024-03-19

**Authors:** BOLORMAA PUREVDORJ, ERIC SUH, ANNE BERIT PETERSEN, YUKI KUWABARA, AYA KINJO, YONEATSU OSAKI, Altanzul Narmandakh, DAVAALKHAM DAMBARDARJAA, PRAMIL SINGH

**Affiliations:** Mongolian National University of Medical Sciences; Loma Linda University Cancer Center; Loma Linda University Cancer Center; Tottori University; Tottori University; Tottori University; National Center for Mental Health, Ministry of Health; Mongolian National University of Medical Sciences; Loma Linda University Cancer Center

**Keywords:** tobacco, adolescent, retail density, school survey, pocket money

## Abstract

**Background:**

The Western Pacific Region has the highest rate of cigarette smoking in the world. In this region, Mongolia has ratified the WHO FCTC treaty and, as part of treaty implementation, has monitored school tobacco use in children using the 2014 Global Youth Tobacco Survey (GYTS) and 2019 GYTS. Our objective was to examine the spatial and economic factors associated with cigarette use in schoolchildren of Mongolia.

**Methods:**

The 2014 and 2019 GYTS are the most recent and comprehensive national surveys of tobacco use in schoolchildren in Mongolia and are cross-sectional, stratified, multi-stage cluster surveys of 13–15 year-old schoolchildren (7,298 in 2014, 4,146 in 2019) selected from urban and rural schools. For each survey, we conducted logistic regression modelling to examine whether spatial (proximity of cigarette sales to schools), economic (pocket money available to school children), and other environmental/contextual factors were predictors of cigarette use (all, single sticks) in schoolchildren.

**Results:**

We found that 1)the prevalence of vendors selling cigarettes near schools increased from 65% in the 2014 GYTS to 94% in the 2019 GYTS, 2) sales of cigarettes near schools were associated with increased current smoking of all cigarettes and this effect increased from a marginal 31% increase in odds in 2014 (OR [95% confidence interval(CI)]=1.31 [0.99, 1.73]) to a 416% increase in odds in 2019 (OR [95% CI]=5.16[3.31, 8.05]), 3) sales of cigarettes near schools were associated with a substantial increase in odds of smoking single cigarettes in 2014 (OR [95% CI]=1.87 [1.14,3.06]) and in 2019 (OR[95% CI]=2.70 [1.42, 5.12]). We also found that smoking of all cigarettes was higher when student pocket money exceeded the price of the cigarette pack (approximately 1.8 USD) and also when parents and/or peers were smokers.

**Conclusions:**

Despite the 2012 National Tobacco Laws banning sales of cigarettes and single cigarettes to schoolchildren near schools, the most recent national surveys (2014–2019) have shown that these sales are increasing. We provide new findings indicating that despite the higher pricing of cigarette packs (relative to the region), illicit sales of single cigarettes are targeting schoolchildren near their schools.

## INTRODUCTION

The WHO has reported that the Western Pacific Region (WPR) has the highest regional rates of tobacco use in the world [1]. For WPR smokers of manufactured cigarettes, this trend is primarily attributable to a 10-fold increase in the prevalence of cigarette smoking in males that starts during their school-age years and results in a 30–50% prevalence of cigarette smoking by the fourth decade of life [1]. In Mongolia, which is located in the WPR, the latest WHO Global Youth and Tobacco Survey (GYTS) data from 2019 indicate that approximately 14% of schoolchildren (21% boys; 6.9% girls) have started using tobacco in any form [2].

In Mongolia, the WHO Framework Convention on Tobacco Control (WHO FCTC) treaty was ratified in 2003. This treaty resulted in the drafting, passage, and implementation of the 2005 Tobacco Control Law, which increased the tax on cigarettes and banned some forms of advertising. Despite the new laws passed in 2005 and further increased in 2012, the prevalence of cigarette smoking in schoolchildren has remained high (over 10%) in Mongolia [2]. This is occurring in Mongolia despite tobacco control laws that ban the following: 1) the sale of cigarettes to persons under the age of 21, 2) the sale of single cigarettes to the population, and 3) the sale of cigarettes within 500 meters of schools. Some of the prevailing trends in cigarette smoking among schoolchildren have been attributed to a lack of enforcement of current tobacco control laws and a lack of resources for tobacco policy implementation at the local level [3]. Considering the high rate of tobacco use documented in the 2019 GYTS survey, it is important to note that findings from other nations indicate that there are social and contextual factors that are strong predictors of youth smoking [4]. Additionally, more investigations are needed to determine how the density of tobacco vendors near schools and pocket money are associated with youth smoking [5].

In the present study, our overall objective is to examine the spatial and economic proximity of tobacco sales to schoolchildren in Mongolia. Our analyses of a national sample of school aged children will provide insight into the level of enforcement of tobacco laws that should restrict the sales of tobacco to children at locations near their schools. Our specific aims include the following 1) To determine whether cigarette vendors (in packs, single cigarettes) near their school influenced a child’s current cigarette use and single cigarette use during the past 30 days, 2) To determine whether a child’s access to pocket money influenced their current cigarette use (in packs, single cigarettes) in the past 30 days. Other contextual factors (peer and family smoking) will also be considered as exposures and potential confounders.

## METHODS

### Study Population

In 2014 and 2019, the GYTS survey was conducted in Mongolia by the National Public Health Institute and WHO Mongolia. The US Centers for Disease Control provided training and advisement on GYTS sampling and questionnaire design in 2014 and 2019.

The sampling methods used for GYTS 2014 and 2019 have been extensively described [2]. Briefly, GYTS sampling in 2014 and 2019 was conducted through a stratified multi-stage cluster sampling where the primary sampling unit was the school (all schools containing grades 7–9). The sampling frame was based on the census and included a national sample of school aged children in Mongolia. The samples were stratified into urban and rural regions. In the first stage of sampling, schools were selected within each strata using a probability proportional to the enrollment size. All students in the selected classes were eligible to participate in the survey. The response rate was 92.3% in 2014 and 92.1% in 2019. This process yielded a total of 7,298 participants in 2014, and 4,146 in 2019.

### Global Youth Tobacco Surveys in 2014 and 2019

The Global Youth Tobacco Survey was self-administered and consisted of 75 multiple choice questions [2]. The questionnaire collected information on a number of tobacco related categories such as: demographics, weekly available pocket money, tobacco use, smokeless tobacco use, access to and availability of tobacco, exposure to tobacco advertisements, susceptibility to smoking, and social and familial contextual factors.

### Statistical Analysis

All the statistical analyses were conducted using SAS-callable SUDAAN (version 9.0), which allowed for a Taylor linearized estimate of variance that accounted for the stratified multi-stage sampling. Using variance inflation factors, variance was increased in all analyses to account for the increased within cluster homogeneity of our clustered sample population. The variables included in the analysis of this study were age, gender, weekly pocket money, current cigarette use, current single cigarette use, parental smoking, friends smoking, and the presence of cigarette vendors near school.

Prevalence and 95% confidence intervals were obtained for current cigarette use and current single cigarette purchases among current cigarette smokers. The same variance estimation methods were used for logistic regression models. Logistic regression analysis was conducted to determine associations between possible predictor variables (pocket money, parental smoking, friends smoking, and the presence of cigarette vendors near schools) and current cigarette use and single cigarette use. Since missing values occurred for less than 10% of the subjects our main analysis excluded the missing values. A sensitivity analysis was performed to examine the effect of multiple imputation and the results did not appreciably change.

Ethics Statement.IRB approval and determination for this secondary analysis of the GYTS study was given by the Institutional Review Board of Loma Linda University (IRB #5170182).

## Results

In Table 1, we provide the demographic, economic, and tobacco use characteristics of the schoolchildren in the GYTS 2014 and 2019. We found that the prevalence of current cigarette smoking (4.8% vs. 4.7%), current tobacco use (18.9% vs, 16.1%), and single cigarette smoking (38.7% vs. 35.7%) was similar across the 2014 and 2019 surveys. Trends in age, gender, parental smoking status, and friends smoking status were also similar across the 2014 and 2019 surveys. The prevalence of cigarettes sold near the school was higher in 2019 (94.3%) than in 2014 (65.3%).

We ran logistic regression models (not shown in the tables) that related cigarette smoking as an outcome variable to the independent demographic, economic, and tobacco use variables in Table 1. We found that older age (OR [95% confidence interval (CI)] per year = 1.76 [1.54, 2.01] in 2014 and 1.54 [1.30,1.84] in 2019) and male gender (OR [95% CI] = 3.02 [2.17, 4.21] in 2014 and 8.66 [5.0, 15.01] in 2019) were associated with higher odds of cigarette smoking.

In Table 2, we present the age and gender-adjusted associations between the economic variables and contextual factors. For economic variables, we found in Table 2 that when cigarettes were sold near schools, children were more likely to be current cigarette smokers - an effect that increased from a marginally significant 31% increase in 2014 to a significant increase of 416% in 2019. Additionally, children who were given more pocket money (> 1 USD) reported higher rates of smoking. The contextual factors of parental smoking and peer smoking were also strongly and significantly associated with current smoking.

We also ran the same logistic regression models among cigarette smokers and classified single cigarette smoking as the outcome. We found that the sale of cigarettes < 500 meters from a school was more strongly associated with student smokers choosing single cigarettes than with choosing packs (OR [95% CI] = 1.85 [1.19, 2.89] in 2014 and 2.70 [1.42, 5.12]). Figure 1 shows that when cigarettes are sold near schools, more than half of the student smokers choose single cigarettes (the prevalence of student smokers choosing single cigarettes [95% CI] = 54.7 [45.2, 64.1] when cigarettes are sold near schools, and 30.9 [21.3, 40.5] when cigarettes are not sold near schools).

## Discussion

Our study focused on the spatial and economic proximity of cigarette packs and single cigarette sales to schoolchildren in Mongolia. We conducted an analysis of the two most recent national surveys of school children in Mongolia (the Global Youth Tobacco Survey (GYTS) in 2014, and the GYTS in 2019) that were conducted since the passage of Mongolian national tobacco control laws from 2005 to 2012. Our main findings indicated the following: 1) the sales of cigarettes near schools increased from 65% in 2014 to 94% in 2019, 2) sales of cigarettes near the schools were associated with increased current smoking of all cigarettes and single cigarettes, and 3) increased weekly pocket money (approximately 1 USD or greater) was associated with increased cigarette smoking. Similar to many other GYTS survey reports from other low and middle-income countries, we also found evidence that the well-known contextual factors of parental smoking [6] and peer smoking [7] strongly influence smoking by schoolchildren in Mongolia.

### Proximity of Cigarette Vendors to Schools and Cigarette Smoking in School Children

Our GYTS findings from Mongolia show a strong and consistent relationship between the spatial proximity (< 500 meters) of cigarette vendors to schools and the higher prevalence of schoolchildren who currently smoked tobacco in 2014 and 2019. It is most alarming that the prevalence of vendors near schools markedly increased from 65% in 2014 to 94% in 2019. These 2019 findings from Mongolia are similar to the environment in urban Indonesia where 96.8% of all schools had tobacco vendors operating within 250 meters of the school [8]. A similar effect remains for all of Indonesia [9]. Indonesia is known for having one of the highest rates of youth smoking in the world [9].

GYTS findings from other nations also indicate the harms of selling cigarettes near schools. In Egypt, 2014 GYTS findings indicate that current smoking by school children was 50–60 fold higher in those attending schools near tobacco vendors (OR = 52.7 (95% CI = 49.2–56.4) and in those attending schools in areas with no local ordinances against the sale to minors (OR = 65.7, CI = 60.5–71.4) [10]. In Nigeria, the 2015 GYTS findings show a similar trend in the proximity of cigarette vendors to schools and higher odds of schoolchildren being current smokers [11]. In 16 African countries (GYTS), youth were less likely to smoke in areas where it is more difficult to purchase cigarettes [12].

Our findings from Mongolia are also broadly concordant with the findings from regions indicating higher prevalence of current smoking in youth in regions with a higher spatial density of tobacco retail outlets. For example, this trend has been shown in GYTS analyses of 16 African nations [12], and also in nations of Southeast Asia [8].

### Proximity of Cigarette Vendors to Schools and Smoking of Single Cigarettes by Student Smokers

Our most recent 2019 data from Mongolia also provide new insight that during the increased sales of cigarettes near schools, vendors appear to be targeting schoolchildren with sales of cheaper, illegal single cigarettes. We found that when vendors were selling cigarettes near school, 170% of the students were smokers of single cigarettes. We also found that when cigarettes were sold near school, more than half of the students who smoked cigarettes smoked single cigarettes (Fig. 1).

We posit that this is likely a method to avoid the trend we found in the Mongolia GYTS, where less than 20% of the children have sufficient weekly pocket money to purchase a cigarette pack (Table 1). Our findings agree with the GYTS findings of Sun et al. from over 140 countries, who reported that the rate of single cigarette smoking was 37% and that the effect is particularly evident in Asia [13]. For example, high rates of single cigarette smoking among student smokers have been reported in GYTS reports from the Philippines (89%) [14], Timor-Leste (39%) [15], and Tunisia (39.4%) [16]. Interestingly, in Timor-Leste, over half (51.6%) of the students indicated that cigarette sellers were close to their school [15].

### Association between Pocket Money given to Schoolchildren and Cigarette Use

Our findings indicating that higher levels of available pocket money given to school children in Mongolia were associated with higher rates of cigarette smoking are concordant with trends from high-, middle-, and low-income countries. In Italy, E Lozza, CM Jarach, G Sesini, E Marta, A Lugo, E Santoro, S Gallus and HL Committee [17] reported that teenagers who spent more than 10€ every week smoked more frequently. According to multivariable models, similar statistically significant positive associations were found in Canada and China [18, 19]. In Bosnia, a positive association was also found between student income and smoking in an analysis of the Global Health Professional Students survey [20]. For low and middle income countries, GYTS findings from more than 16 African Nations [12] [21], and a separate analysis from the China National Youth Tobacco survey all confirmed a positive association between pocket money given to school children and current smoking status [22, 23].

Taken together, our findings from our study of Mongolia and nations from several other WHO regions (African, Americas, European, and Western Pacific) indicate that the implementation of WHO FCTC measures to increase the price of cigarette packs beyond the weekly spending money of schoolchildren, adolescents, and adult students could be effective in preventing the initiation of tobacco. This is directly supported by GYTS analyses of the negative association indicating that lower cigarette pricing is associated with higher levels of youth smoking [12].

Implications for WHO FCTC Policy Implementation and Enforcement of the National Tobacco Control Law in Mongolia

Our findings from the two most recent GYTS surveys of Mongolia (2014, 2019) indicate a lack of enforcement of the 2012 [24] National Tobacco Control law: banning 1) sales of cigarettes to persons under age 21 (article 2, section 6 (6.7.7)), 2) sales of cigarettes within 500 m of a school(article 2, section 6 (6.7.14)), and 3) sales of single cigarettes to all persons (article 2, section 6(6.7.8)) [25].

Our findings highlight the need for a multi-sectoral approach to tobacco control that involves the Ministry of Health, Ministry of Justice, and Ministry of Education. Currently violation of these laws are a minor offence with low fines. [26].

### Limitations

Several limitations of the 2014 and 2019 GYTS in Mongolia should be noted. First, the GYTS is self-reported and thus subject to recall bias. Second, the GYTS does not collect information such as household income, and school tobacco prevention programs, was not collected for the GYTS, which may be important factors that were not adjusted for in the analysis. Third, this study used cross sectional data; therefore, the temporal relationship between outcomes and factors cannot be determined. It is noteworthy that these cross-sectional measures occurred after the implementation of the 2012 National Tobacco Control Law and so our findings do provide a longitudinal perspective on the effect of the law during the subsequent 7 years.

## Conclusions

Our findings from surveys of school children in 2014 and 2019 indicate the need for greater enforcement of national tobacco laws on sales of cigarettes and single cigarettes to minors near schools. Despite efforts to increase the price ofcigarette packs, vendors are targeting school children near their schools with low priced single cigarettes.

## Figures and Tables

**Figure:**
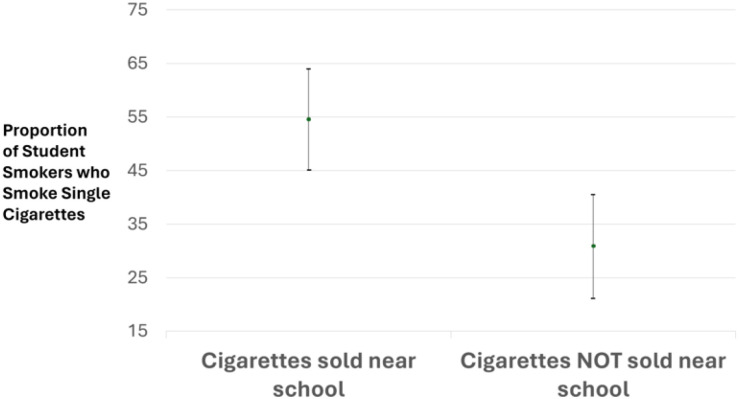
Prevalence and 95% confidence interval of student smokers who smoke single cigarettes is given by proximity of cigarette vendors to their school in Mongolia (Global Youth Tobacco Survey 2019).

**Table 1:** Demographic, Economic, and Tobacco use variables from school-aged children enrolled in the Global Youth Tobacco Survey (GYTS) of Mongolia.

Variable	GYTS 2014 (n=7298)	GYTS 2019 (n=4146)
	Prevalence (95% Cl)	Prevalence (95%, Cl)
**Age (years)**		
*Age 10–11*	*0.3 (0.1–0.5)*	*0.3 (0.2–0.4)*
*Age 12*	*4.9 (3.1–6.7)*	*7.9 (5.8–9.9)*
*Age 13*	*39.5 (34.9–44.1)*	*32.3 (28.7–36.0)*
*Age 14*	*27.6 (24.3–30.9)*	*34.0 (31.3–36.7)*
*Age 15*	*17.1 (14.4–19.8)*	*22.1 (18.7–25.5)*
*Age 16*	*8.6 (6.5–10.7)*	*3.2 (1.9–4.5)*
*Age > 17*	*2.01 (0.9–3.1)*	*0.26 (0.1–0.4)*
**Gender**		
*Female*	*48.5 (46.8–50.2)*	*50.2 (48.0–52.4)*
*Male*	*51.5 (49.8–53.2)*	*49.8 (47.6–52.0)*
**Current Cigarette User**	*5.9%*	*4.4%*
**Current Tobacco User**	*19.6%*	*15.4%*
**Single Cigarette Smokers**	*41.5%*	*35.4%*
**Weekly Pocket Money**		
*< 0.14USD*	*12.5 (11.0–14.0)*	*12.5 (10.1–14.9)*
*0.14 to 0.29 USD*	*13.6 (12.3–14.9)*	*9.8 (7.8–11.7)*
*0.30 to 0.43 USD*	*25.0 (23.2–26.9)*	*21. 4 (19.2–23.7)*
*0.44 to 0.57 USD*	*13.0 (12.0–14.0)*	*14.8 (13.0–16.6)*
*0.58 to 0.71 USD*	*9.6 (8.8–10.5)*	*9.0 (8.0–10.0)*
*0.72 to 0.86 USD*	*10.0 (8.7–11.3)*	*12.0 (11.1–12.9)*
*> 0.86 USD*	*16.3 (14.1–18.4)*	*20.5 (16.9–24.2)*

**Table 2:** Multivariable logistic regression models relating demographic and economic variables to current smoking among school aged children enrolled in the Global Youth Tobacco Survey (GYTS) in 2014 and 2019.

Variable	GYTS 2014	GYTS 2019
	adjusted OR (95% Cl)	adjusted OR (95% Cl)
**Cigarettes sold near school**		
*Yes*	*1.31 (0.99,1.73)*	*5.16 (3.31,8.05)*
*No*	1.0 (referent)	1.0 (referent)
**Weekly Pocket Money given to Student**		
*< 0.14USD*	*1.00*	*1.00*
*0.14 to 0.29 USD*	*0.84 (0.48, 1.48)*	*1.12 (0.62,2.03)*
*0.30 to 0.43 USD*	*0.85 (0.51,1.41)*	*1.57 (0.92,2.68)*
*0.44 to 0.57 USD*	*1.34 (0.75,2.42)*	*1.35 (0.75,2.44)*
*0.58 to 0.71 USD*	*1.42 (0.83,2.45)*	*1.85 (0.94,3.67)*
*0.72 to 0.86 USD*	*1.55 (0.95,2.52)*	*1.50 (0.73,3.06)*
*> 0.86 USD*	*2.49 (1.49,4.17)*	*1.42 (0.73,2.77)*
**Friends Smoke**		
*None of them*		
*Some*	*5.58 (3.24,9.61)*	*8.25 (5.39,12.63)*
*Most*	20.47 (11.67,35.92)***	16.92 (8.17,35.04)**
*All*	18.41 (8.90,38.05)***	12.76 (5.34,30.50)*
**Parents Smoke**		
*None*	*1.00*	
*Both*	*3.72 (2.32,5.96)*	*3.22 (2.05,5.07)*
*Father only*	*1.31 (1.01,1.70)*	*1.40 (0.98,2.02)*
*Mother only*	*5.60 (3.07,10.21)*	*1.48 (0.48, 4.59)*
